# Month of birth and outdoor temperature after birth predict childhood atopic diseases in Finland

**DOI:** 10.1111/pai.70118

**Published:** 2025-06-02

**Authors:** Juha Luukkonen, Heta Moustgaard, Hanna Remes, Pekka Martikainen

**Affiliations:** ^1^ Helsinki Institute for Demography and Population Health University of Helsinki Helsinki Finland; ^2^ Max Planck–University of Helsinki Center for Social Inequalities in Population Health Helsinki Finland; ^3^ Finnish Social Insurance Institution Helsinki Finland

**Keywords:** allergic rhinitis, asthma, atopic disease, eczema, food allergy, month of birth, outdoor temperature after birth

## Abstract

**Background:**

Previous evidence on season‐of‐birth differences in atopic diseases is partially mixed, and the etiology behind them is not well understood. For example, outdoor temperature may be an important modifier of the association but has been previously neglected.

**Methods:**

We assess how the month of birth is associated with medication use for atopic diseases at ages 0–15 and how outdoor temperatures after birth modify these associations for 0.55 million Finns born during 1995–2004. We used Finnish register data on purchases of medications used for allergic rhinitis, eczema, asthma, and food allergies. We predicted month‐of‐birth associations with medication use using extensive controls for observed and unobserved confounders and assessed effect modification by 3‐month mean outdoor temperature after birth.

**Results:**

Approximately half of the children had at least one purchase of medication used for atopic diseases. For children born in spring or summer, the probability of medication use was moderately lower than for children born in the autumn or winter. Among children born in the autumn or winter, exposure to the coldest outdoor temperatures in the first 3 months of life was associated with a nearly 10‐percentage‐point increase in the risk for medication use compared with the warmest temperatures.

**Conclusions:**

Behind the moderate overall associations between month of birth and childhood atopic diseases, there was notable variation by environmental conditions after birth, with cold weather after birth being particularly harmful. Future studies should assess what specific exposures do the outdoor temperatures affect, and in turn how they affect the development of atopic diseases.

**Key message**

Using Finnish population register data, this study showed that behind modest month‐of‐birth differences on childhood atopic diseases, there was notable variation by outdoor temperature after birth. Especially cold weather after autumn or winter birth considerably increased the risk for atopic disease medication use. Future assessment of environmental risk factors related to season of birth should focus on what specific exposures do the outdoor temperatures affect, and in turn how they affect the development of specific atopic diseases.

## 1 INTRODUCTION

Atopic diseases have rapidly become more common in recent decades.^1^ As these diseases usually start in early childhood and are partly influenced by early‐life environment, a better understanding of both protective and risk‐inducing environmental factors is warranted.^1^ Some studies have found a decreased likelihood of developing atopic diseases for children born in the spring or summer in the northern hemisphere and Australia.^2^, ^3^, ^4^, ^5^, ^6^, ^7^, ^8^, ^9^, ^10^ However, evidence on the association between season of birth and atopic diseases is somewhat inconsistent, as shown in a meta‐analysis on eczema and a large multi‐center study on allergic rhinitis.^2^, ^11^ Also, birth in the autumn or winter is generally associated with an increased likelihood of asthma,^6^, ^7^, ^8^, ^10^ with some evidence of an increased risk of asthma also among children born in the summer in a warmer climate.^12^

The role of environmental modifiers in the etiology of atopic diseases is still unclear.^13^ For example, among the mechanisms behind the atopic disease, differences by season of birth might be the still developing epidermal functioning of infants and/or beneficial exposure to microbiota, both of which might be compromised by being born during winter due to, for example, increased risk of respiratory infections, decreased indoor humidity, and increased staying indoors.^1^, ^14^, ^15^, ^16^ The mixed findings from previous studies on the relationship between season of birth and atopic diseases may be due in part to overlooking the variability in seasonal changes. Furthermore, the data from prior studies are often based on small samples that lack potential confounders.^2^

Using Finnish population register data linked with prescription medication use data, we assessed how the association between month of birth and childhood atopic diseases differs by spatiotemporal variation in seasons. Finland is a Nordic country with four seasons and large seasonal variation in outdoor temperatures. Finland ranges between 60° N and 70° N parallels, and thermal spring starts later and autumn earlier in northern Finland, making the winters longer than in Southern Finland.^17^ Finland thus provides a suitable setting for research on how environmental conditions at time of birth may affect the likelihood of childhood atopic diseases.

We assessed the month‐of‐birth associations in the general population with comprehensive measures for atopic diseases. We considered purchases of antihistamines, eczema medication, asthma medication, and epinephrine between ages 0 and 15. We controlled for several potential confounders including regional characteristics, family demographic and socioeconomic characteristics, and family history of atopic diseases that are known risk factors for atopic diseases and may be associated with the timing of birth.^18^ Unobserved familial confounding was assessed with sibling fixed‐effects regressions.

We further assessed whether the relationship between month of birth and atopic diseases differs by mean outdoor temperature in the first 3 months after birth, for example, how being born during a warm winter is associated with the probability of atopic disease medication compared with a cold winter.

## 2 METHODS

### 2.1 Finnish register data

The study population consisted of children born in 1995–2004 identified from the longitudinal population register of Statistics Finland. This individual‐level register contains annual information on the full population residing in Finland with linkages between biological family members. The data were linked with individual‐level information on medication purchases from the national prescription register maintained by the Social Insurance Institution of Finland^19^ and information on mode of delivery, birth weight, and gestational age from the Medical Birth Register.

We included individuals born and resident in Finland throughout ages 0–15 years (*N* = 554,322). We excluded children who emigrated (*n* = 20,015) or died (*n* = 3275) before age 15 years. In the within‐family analyses, we restricted the sample to children with at least one full sibling to compare with *N* = 339,457.

The study has been approved by Statistics Finland Board of Statistical Ethics (TK‐53‐1490‐18) and the Social and Health Data Authority Findata (THL/2180/14.02.00/2020). The register data are originally collected for administrative and statistical purposes, and no informed consent was thus required. The legal basis for processing this kind of personal information is scientific research as stated in the Finnish Personal Data Act (523/1999) and the EU General Data Protection Regulation (GDPR). The national act gives the possibility to process special category data (as stated in the Art. 9 of the GDPR) for scientific research carried out in public interest.

### 2.2 Outcome: purchases of medications used in the treatment of atopic diseases

We used publicly reimbursed purchases of prescription medications as measures for common childhood atopic diseases (allergic rhinitis, eczema, asthma, food allergies, and severe allergic reactions). All residents of Finland are entitled to at least partial reimbursement for most prescription‐issued medication provided directly at pharmacies.^19^ The medications were identified and categorized according to the Anatomic Therapeutic Chemical (ATC) classification (Table S1). We measured purchases of each medication type (antihistamines, eczema medication, asthma medication, and epinephrine) with an indicator for at least one purchase by the 15th day of the month of the child's 15th birthday and a pooled outcome of any type of atopic disease medication.

### 2.3 Outdoor temperature data

We used open data from the Finnish Meteorological Institute^20^ consisting of month‐of‐year average outdoor temperatures measured at different weather stations (*n* = 78) from 1995 to 2004. With the coordinates of each weather station, we matched the child's home municipality at birth with the nearest weather station, excluding some stations operating in highly elevated places. The average municipal distance to a weather station was 26 kilometers. The average outdoor temperatures across the first 3 months of life were modeled as cubic, as there may be nonlinear associations between medication and outdoor temperatures. Individuals with missing observations on monthly temperature were excluded from the analyses (*n* = 2791, 0.5% of total).

### 2.4 Observed confounders

We controlled for several confounders measured in the year of birth unless otherwise specified. We controlled for sex and birth year (categorical) of the child and parental immigrant status (at least one parent born abroad, yes/no), which are risk factors for atopic diseases.^21^

Geographical area (NUTS3 regions) controlled for potential regional differences in access to healthcare and prescription practices.^11^, ^22^, ^23^ We also controlled for the level of urbanicity of the municipality of residence, as less biodiverse and more polluted urban environments are associated with increased rates of asthma and allergies than rural environments.^24^, ^25^, ^26^, ^27^

The highest parental education (basic, secondary, or tertiary) and household income decile based on annual disposable income per household consumption unit were included to control for possible socioeconomic differences in treatment‐seeking behavior and affordability of medication.

We also included variables measuring birth order and the number of biological siblings, as higher birth order and a higher number of siblings have been observed to reduce the likelihood of atopic diseases.^28^

Finally, we further controlled for mode of delivery (Caesarean section vs. other), as caesarean sections are associated with increased risk of some atopic diseases, as well as birth weight (grams, squared) and gestational age (days, squared).^29^ We also controlled for parental medication purchases (none vs. at least one purchase of the measured medications while the child was aged 0–15 years) as separate dummies for each medication type to address genetic susceptibility for atopic diseases.

### 2.5 Analyses

We used linear probability models with heteroscedasticity robust standard errors to predict the probability of medication purchases by month of birth in separate models for specific medication types and for any atopic disease medication. We present our results as absolute differences in the probability of medication purchases compared to those born in January. The basic model controlled for sex and birth year of the child and parental immigrant status. The fully adjusted models controlled for region and urbanicity of residence, household income and parental education, sibship size and birth order, mode of delivery, birth weight, gestational age, and parental medication purchases. To further control for unmeasured familial confounding, we used sibling fixed‐effects models. These models are based on comparisons of full siblings with different months of birth and control for all exposures shared by siblings by design. Such shared unobserved time‐invariant confounders can relate to genetic susceptibility to atopic diseases, physical exposures in the growth environment, or healthcare‐seeking behavior within the family.

Next, we assessed whether the association between month of birth and purchases of medication used in the treatment of atopic diseases differed by the average outdoor temperature in the 3 months after birth. For the sake of simplicity, we present the temperature analysis results for the pooled atopic disease medication category and show the results of specific medication types as Data S1.

We conducted several sensitivity analyses assessing the robustness of the results. First, we tested the sex of the child as a moderator of month of birth, as there are sex differences in immune responses that develop during early childhood.^30^ Second, to better capture chronic conditions, we restricted the outcomes to those with purchases in at least three calendar years per medication type. Furthermore, we used special reimbursement entitlement for asthma medication by age 15 as an alternative diagnostics‐based measure for asthma. The special reimbursement entitlement is granted by the Social Insurance Institution for individuals with chronic asthma as confirmed by a medical evaluation including pulmonary function tests.^31^

Finally, we tested the validity of the outdoor temperature exposure by varying how many months were included in the mean temperature after birth. Besides the 3‐month average, we used two‐ and one‐month averages.

## 3 RESULTS

For the birth cohorts 1995–2004, approximately half had at least one purchase of any medication used in the treatment of atopic diseases between ages 0 and 15 years. Over a third of the population purchased antihistamines, and 32% purchased eczema medication. Purchases of asthma medication (18%) and epinephrine (3%) were less common. In general, the children born in the summer had the lowest risk for any type of atopic disease medication, but there were notable differences by medication type (Table 1, sibling population shown in the Table S2).

**TABLE 1:** Prevalence of purchases of medication used for atopic diseases for children aged 0–15 years (%) by month of birth. Finnish birth cohorts 1995–2004.

Month of birth	*N*	Antihistamines	Eczema medication	Asthma medication	Epinephrine	Any medication
Jan	46,171	37.9	31.8	17.9	2.9	55.9
Feb	43,081	38.4	31.4	18.1	3.0	55.8
Mar	48,548	38.1	31.0	17.8	3.0	55.7
Apr	47,774	37.7	30.7	17.4	2.5	55.5
May	47,769	36.5	30.7	16.6	2.4	54.8
Jun	47,123	36.1	30.5	16.8	2.3	54.4
Jul	49,332	36.0	31.4	17.0	2.4	55.0
Aug	48,060	36.1	31.9	17.3	2.4	55.3
Sep	46,835	36.0	32.4	17.6	2.5	55.2
Oct	45,113	36.0	32.6	17.9	2.8	55.5
Nov	41,822	36.9	32.3	18.3	3.0	55.8
Dec	42,694	37.6	32.1	18.2	3.1	55.6
Total	554,322	36.9	31.6	17.6	2.7	55.3

*Note:* 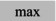, Maximum; 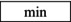, Minimum.

For antihistamines, the prevalence was lowest for children born between June and October (range 36.0–36.1%) and highest for children born in winter months and early spring (range 36.9–38.4%).

For eczema medication, the prevalence was lowest for children born between March and June (range 30.5–30.7%) and highest for children born between August and January, with October peaking at 32.6%. For asthma medication, the prevalence was lowest for children born during May, June, and July (16.6–17.0%). The prevalence was highest among children born between November and February (approximately 18.0%).

The prevalence for epinephrine was lowest among children born from May to August (range 2.3–2.4%) and highest among children born from October to March (range 2.8–3.0%).

The relative differences between months of birth were greatest for epinephrine (26%) and ranged from 6 to 9% for the more common medications. As the seasonality patterns differed across medication types, the any medication category had the smallest differences across months of birth (3%).

The measured risk factors for atopic diseases showed either no or modest variation according to the month of birth (Tables S3 and S4).

**FIGURE 1:**
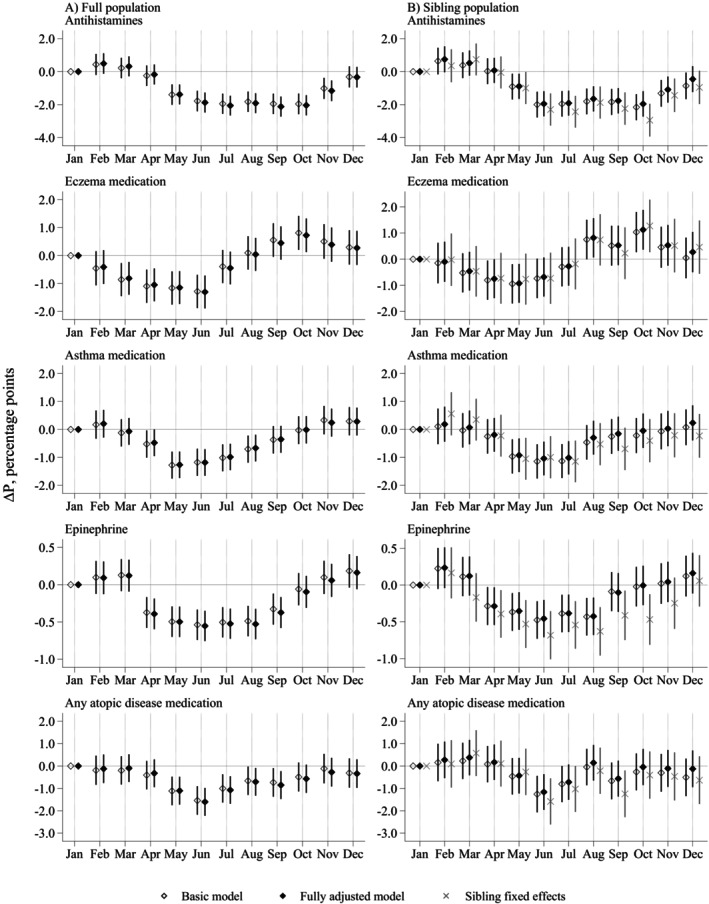
Estimated difference in probability (∆*P*, percentage points) of purchases of medication used for atopic diseases at ages 0–15 years by month of birth in reference to January. Results from (A) full population models (*N* = 554,322) and (B) sibling population models (*N* = 339,457) with 95% confidence intervals. The basic model is adjusted for child's sex, birth year, and immigrant background. The fully adjusted model is further adjusted for region and urbanicity of residence; household income and parental education; sibship size and birth order; mode of delivery; and parental medication purchases. The sibling fixed‐effects model is adjusted in the same way as the fully adjusted model with the addition of sibship‐specific dummy variables.

In the full population (Figure 1, panel A), the probability of atopic medication purchases was higher among children born in the autumn or winter, even after adjusting for a wide range of potential confounders. However, seasonal patterns differed by medication type. For antihistamines, the probabilities were higher among those born from winter to early spring, whereas for asthma, the risks were higher already among those born in the autumn. For epinephrine, the higher risks further extended to births in early autumn to mid spring. In contrast to other medications, the probability of eczema purchases increased already among those born in late summer.

In the sub‐population consisting of siblings (panel B), the medication prevalence was slightly lower compared with the full population (Table S2), which is in line with previous findings showing that having siblings is protective against atopic diseases.^28^ The associations between month of birth and atopic disease medication were nonetheless highly similar, even in the sibling fixed‐effects models controlling for shared environmental and genetic risk factors for atopic diseases.

**FIGURE 2:**
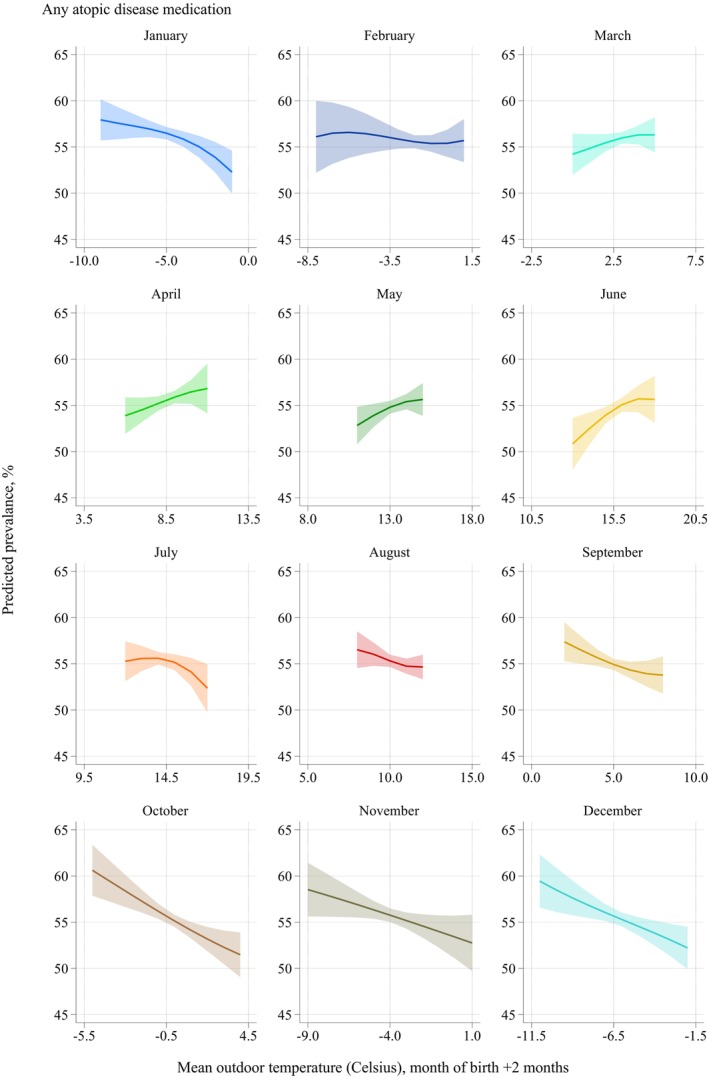
Moderation by outdoor temperatures after birth. Predicted probability (%) of any childhood purchases of medication used for atopic diseases at ages 0–15 years by month of birth and 3‐month average temperatures after birth. Results from full population models with 95% confidence intervals (*N* = 551,531). Each month of birth was analyzed separately with the 3‐month averages of recorded outdoor temperatures in centigrade based on spatial and temporal variation during 1995–2004. Predictions for the values are within the 90% interquartile range of temperatures for each month. A cubic polynomial of temperature was used. Note the difference in x‐axis values.

We assessed how the 3‐month average outdoor temperature after birth was associated with the risk of medication purchase by each month of birth (Figure 2, see Figures S2–S5 for different medication types).

Among children born between October and January, cold weather in the months following birth was associated with notable increases in the probability of atopic disease medication purchase. For example, among October‐born children, exposure to the coldest temperatures in the first 3 months after birth was associated with a 9‐percentage‐point increase in the atopic disease medication purchases when compared with the warmest temperatures. Outdoor temperature following birth was not associated with medication purchases among February‐born children. Among children born from March to June, warmer weather in the months following birth was associated with a modest increase in the probability of atopic disease medication purchase.

As shown in Figures S1–S4, these associations were clearly observable with antihistamines and eczema medication and somewhat similar for asthma medication. For epinephrine, the outdoor temperature difference associations were largely absent, but the lower probability of medication purchase was still apparent for children born in the summer or spring. The steepest medication‐temperature gradient was observable among October‐born children for all medication types.

According to the sensitivity analyses, seasonality was slightly stronger for boys (Figure S5). When measuring medication purchases in at least 3 years per medication type and special reimbursement rights for asthma medication to capture more chronic cases, the results were highly similar or slightly stronger than the main results (Figures S6–S11). Finally, the associations were similar regardless of whether outdoor temperature was measured over a one‐, two‐, or three‐month period after birth (Figure S12).

## 4 DISCUSSION

Using population‐wide data, we showed month‐of‐birth variation in the risk of atopic disease medication purchase by the age of 15 years, with a lower probability for children born in the spring or summer in contrast to children born in the autumn or winter. While similar seasonality has been observed in previous studies on the Northern Hemisphere,^2^, ^3^, ^4^, ^5^, ^6^, ^7^, ^8^, ^9^ we showed that the associations were independent of familial background, which has been largely neglected in previous studies. Although previous studies have mostly assessed specific atopic disease outcomes, we also showed notable seasonality differences by different types of atopic disease medication, suggesting that there may be several mechanisms driving the development of atopic manifestations.

While the overall differences between seasons of birth were modest, there were notable differences by outdoor temperature variation in the months following birth. For children born in the autumn or winter, exposure to colder outdoor temperature was associated with notable increases in the probability of later atopic disease medication purchases. Conversely, warmer outdoor temperatures after birth were associated with slightly increased atopic disease medication purchase probability for children born in March to June. The aforementioned associations were mostly observed with antihistamines and eczema medication and to some degree with asthma medication. The temperature patterns with epinephrine were less consistent—likely due to the rarity of the outcome. Overall, the results related to outdoor temperature variation may explain why the previous cross‐country findings on seasonal variation have been mixed^2^, ^11^: The contextual climate is relevant in the development of atopic diseases.

### 4.1 Potential mechanisms

Based on our results, outdoor temperature following birth seems to be an important environmental factor influencing the development of atopic diseases over and above the season of birth, but the causal pathways and underlying mechanisms remain unclear. There are several potential contributors.

According to the epithelial barrier hypothesis, a number of allergens, pathogens, and environmental toxins can damage the epithelial barrier, and defective epithelial barrier functioning has been demonstrated with a variety of atopic diseases.^32^, ^33^ As the epithelial barrier of newborns and infants is still adapting to the postnatal environment,^14^ seasonal variation in early‐life exposures that disrupt the epithelial barrier could be notable in the development of atopic diseases. For example, cold outdoor temperatures increase the risk of respiratory infections,^34^ early‐life exposure to which has been observed to increase the risk of developing asthma.^7^, ^35^ This is in accordance with our finding that being born in cold winter months was associated with higher asthma medication risk.

Additionally, airborne pollution exposure might increase from the wintertime heating season or inversions in outdoor temperature creating smog episodes,^36^ and could also contribute to epithelial barrier impairment. Furthermore, for winter‐born infants, exposure to lower indoor humidity during the first months of life has been observed to correlate with impaired epithelial barrier functioning, resulting in increased rates of childhood eczema.^14^, ^23^ Our observation of lower outdoor temperature being associated with increased probability of eczema medication was in line with this, as in Finland, indoor humidity can be particularly low once sub‐zero outdoor temperatures are reached.^37^

Conversely, warmer outdoor temperatures during spring and summer are associated with longer and more intense pollen seasons for some plants,^38^ and pollen exposure in early childhood is associated with more atopic diseases,^39^, ^40^ which might explain the observed increased risk for antihistamine purchases among children born in March to June who experienced particularly warm weather after birth.

The exposures affected by season could also be beneficial. It has been speculated that for children born in the spring or summer, exposure to more ultraviolet‐B radiation may improve skin‐barrier functions and consequently prevent onset of atopic diseases^14^, ^23^ through decreased inflammation and enhanced epithelial barrier performance.^14^ While ultraviolet‐B exposure may explain some of the seasonal differences, it is unlikely to account for the outdoor temperature differences during winter time when ultraviolet light exposure in Finland is very limited overall.^41^

According to the microbiota hypothesis, early‐life exposure to microorganisms boosts the development of the immune system; less exposure to diverse microbiota in the early‐life environment and consequently in the human body may result in inflammatory responses to usually harmless allergens.^15^, ^16^ The exposure to diverse microbiota among children born in the autumn or winter may be compromised due to spending more time indoors at the start of life. The seasonality of microbiology in built environments is complex and still poorly understood,^42^ but less indoor humidity during the autumn and winter may be associated with seasonal variation of indoor microbiota and consequently with atopic diseases, and indoor transfer of dirt‐associated environmental bacteria is reduced during winter.^43^ Accordingly, winter‐born children have been observed to have a poorer nasal microbiota at age 1 month compared with summer‐born children.^44^ Plant richness in the environment, a potential advantage for children born in the spring or summer, has also been associated with a richer skin microbiota and less atopic diseases among Finnish adolescents.^25^

### 4.2 Strengths and limitations

Longitudinal population‐wide register data allowed assessment of month‐of‐birth purchase differences in specific medication types, measuring the spectrum of atopic diseases, and allowed us to account for several potential confounding factors, including unobserved familial confounding. We were also able to include atopic medication purchases across childhood from birth up to age 15. Furthermore, Finland provides an optimal setting to study how season of birth is associated with childhood atopic diseases, as there is outdoor temperature variation between and within seasons. Given the absence of previous studies on outdoor temperature in seasonal variation, the generalizability of our findings beyond similar climate conditions remains an open question. Overall, little is known about seasonal patterns in atopic diseases in warmer climates.

Our study has its limitations. The reimbursed prescription medication purchases in our data do not fully reflect the prevalence of atopic diseases, particularly in less severe, occasional atopic symptoms. Some antihistamines and eczema medication can be purchased over the counter and are not reimbursed, and some prescription‐only medications are not reimbursed and are thus not included in our data.^45^ However, our measure for asthma medication (combination inhalers and inhaled corticosteroids) is particularly suitable to identify diagnosed asthma.^46^ Furthermore, all asthma medications require a prescription and are reimbursed,^45^ and all epinephrine medications also require a prescription, and a vast majority of products are reimbursed.^45^ While there is a clear incentive to obtain a prescription and reimbursement in cases of long‐term need or need for more potent medication, children with atopic symptoms who were not treated or used non‐reimbursed medications were misclassified as having no symptoms. This may have led to an underestimation of atopic diseases in the population. However, we do not believe this biased the studied associations, as there is no reason to expect this to vary by month of birth or outdoor temperature. Potential family‐level healthcare‐seeking behavior was controlled for by the sibling fixed‐effects models, with little effect on the results.

The sibling fixed‐effects models also controlled for other unobserved family‐level risk factors, such as genetic susceptibility to atopic diseases and physical exposures in the growth environment or healthcare‐seeking behavior, shared by siblings. Finally, pre‐ and postnatal exposures, such as maternal diet, breastfeeding practices, or the risk of respiratory infections, may also vary by season and outdoor temperature, and thus confound our findings. We controlled for gestational age and mode of delivery, but a more in‐depth analysis on the role of in‐utero exposures and maternal behaviors in seasonal patterning of offspring atopic diseases remains an important avenue for future research.

## 5 CONCLUSION

Using population data, we found fewer atopic disease medication purchases among children born in the spring or summer even after adjusting for potential confounders. The varying seasonality patterns across medication types, used in the treatment of different atopic diseases, suggest that there may be several environmental mechanisms involved. Furthermore, for children born in autumn or winter, exposure to colder outdoor temperatures in the months following birth was associated with notable increases in antihistamine, eczema medication, and asthma medication purchases by age 15 years, suggesting considerable variation in how the season of birth is associated with the development of atopic diseases.

## AUTHOR CONTRIBUTIONS

**Juha Luukkonen:** Conceptualization; investigation; writing – original draft; methodology; visualization; writing – review and editing; formal analysis; data curation; project administration. **Heta Moustgaard:** Methodology; writing – original draft; writing – review and editing; supervision; visualization; formal analysis. **Hanna Remes:** Methodology; writing – original draft; supervision; visualization; formal analysis; writing – review and editing. **Pekka Martikainen:** Funding acquisition; conceptualization; writing – review and editing; methodology; writing – original draft; supervision; resources.

## FUNDING INFORMATION

This work was supported by the European Research Council under the European Union's Horizon 2020 research and innovation program (#101019329), the Strategic Research Council (SRC) within the Academy of Finland with grants for ACElife (#352543‐352572) and LIFECON (#345219), the Research Council of Finland profiling grant for SWAN and FooDrug and grants to the Max Planck—University of Helsinki Center from the Jane and Aatos Erkko Foundation (#210046), the Max Planck Society (#5714240218), University of Helsinki (#77204227), and Cities of Helsinki, Vantaa, and Espoo. The study does not necessarily reflect the Commission's views and in no way anticipates the Commission's future policy in this area. The funders had no role in the study design, data collection and analysis, decision to publish, or preparation of the manuscript.

## CONFLICT OF INTEREST STATEMENT

None declared.

### PEER REVIEW

The peer review history for this article is available at https://www.webofscience.com/api/gateway/wos/peer‐review/10.1111/pai.70118.

## Supporting information


Data S1.

## References

[1] Schoos AM . Atopic diseases—diagnostics, mechanisms, and exposures. Pediatr Allergy Immunol. 2024;35(7):e1‐e16. doi:10.1111/pai.14198 39016386

[2] Calov M , Alinaghi F , Hamann CR , Silverberg J , Egeberg A , Thyssen JP . The association between season of birth and atopic dermatitis in the northern hemisphere: a systematic review and meta‐analysis. J Allergy Clin Immunol Pract. 2020;8(2):674‐680. doi:10.1016/j.jaip.2019.10.007 31678290

[3] Mullins RJ , Clark S , Katelaris C , Smith V , Solley G , Camargo CA Jr . Season of birth and childhood food allergy in Australia. Pediatr Allergy Immunol. 2011;22(6):583‐589. doi:10.1111/j.1399-3038.2011.01151.x 21342281

[4] Vassallo MF , Banerji A , Rudders SA , Clark S , Mullins RJ , Camargo CA . Season of birth and food allergy in children. Ann Allergy Asthma Immunol. 2010;104(4):307‐313. doi:10.1016/j.anai.2010.01.019 20408340 PMC2941399

[5] Nilsson L , Bjorksten B , Hattevig G , Kjellman B , Sigurs N , Kjellman NIM . Season of birth as predictor of atopic manifestations. Arch Dis Child. 1997;76(4):341‐344. doi:10.1136/adc.76.4.341 9166028 PMC1717161

[6] Koskinen A , Lemmetyinen R , Luukkainen A , et al. Season of birth affects the risk of adult‐onset asthma in Finland. Allergy. 2023;78(2):555‐558. doi:10.1111/all.15504 36067009 PMC10087432

[7] Almqvist C , Ekberg S , Rhedin S , Fang F , Fall T , Lundholm C . Season of birth, childhood asthma and allergy in a nationwide cohort—mediation through lower respiratory infections. Clin Exp Allergy. 2020;50(2):222‐230. doi:10.1111/cea.13542 31782836

[8] Åberg N . Birth season variation in asthma and allergic rhinitis. Clin Exp Allergy. 1989;19(6):643‐648. doi:10.1111/j.1365-2222.1989.tb02761.x 2598104

[9] Saitoh Y , Dake Y , Shimazu S , et al. Month of birth, atopic disease, and atopic sensitization. J Invest Allergol Clin Immunol. 2001;11(3):183‐187. 11831451

[10] Hänninen R , Murtomäki A , Svärd F , et al. Being born in autumn or winter is associated with asthma and allergic rhinitis in Finland. Clin Transl Allergy. 2024;14(7):e12383. doi:10.1002/clt2.12383 39031968 PMC11259556

[11] Wjst M , Dharmage S , André E , et al. Latitude, birth date, and allergy. PLoS Med. 2005;2(10):e294. doi:10.1371/journal.pmed.0020294 16190778 PMC1240049

[12] Goldberg S , Stein A , Picard E , et al. Does birth season influence the odds for asthma? Large cohort analysis. Pediatr Pulmonol. 2020;55(5):1111‐1115. doi:10.1002/ppul.24677 32032463

[13] Dharmage SC , Lowe AJ , Matheson MC , Burgess JA , Allen KJ , Abramson MJ . Atopic dermatitis and the atopic march revisited. Allergy. 2014;69(1):17‐27. doi:10.1111/all.12268 24117677

[14] Thyssen JP , Zirwas MJ , Elias PM . Potential role of reduced environmental UV exposure as a driver of the current epidemic of atopic dermatitis. J Allergy Clin Immunol. 2015;136(5):1163‐1169. doi:10.1016/j.jaci.2015.06.042 26298230

[15] Wise SK , Lin SY , Toskala E , et al. International consensus Statement on allergy and rhinology: allergic rhinitis: ICAR: allergic rhinitis. Int Forum Allergy Rhinol. 2018;8(2):108‐352. doi:10.1002/alr.22073 29438600

[16] Noverr MC , Huffnagle GB . The “microflora hypothesis” of allergic diseases. Clin Exp Allergy. 2005;35(12):1511‐1520. doi:10.1111/j.1365-2222.2005.02379.x 16393316

[17] Ruosteenoja K , Räisänen J , Pirinen P . Projected changes in thermal seasons and the growing season in Finland. Int J Climatol. 2011;31(10):1473‐1487. doi:10.1002/joc.2171

[18] Buckles KS , Hungerman DM . Season of birth and later outcomes: old questions, new answers. Rev Econ Stat. 2013;95(3):711‐724. doi:10.1162/REST_a_00314 24058211 PMC3777829

[19] Finnish Medicines Agency Fimea Social Insurance Institution . Finnish Statistics on Medicines 2011 . 2012.

[20] The Finnish Meteorological Institute . The Finnish Meteorological Institute's open data . 2024 https://en.ilmatieteenlaitos.fi/open‐data

[21] De Swert LFA . Risk factors for allergy. Eur J Pediatr. 1999;158(2):89‐94. doi:10.1007/s004310051024 10048601

[22] Osborne NJ , Ukoumunne OC , Wake M , Allen KJ . Prevalence of eczema and food allergy is associated with latitude in Australia. J Allergy Clin Immunol. 2012;129(3):865‐867. doi:10.1016/j.jaci.2012.01.037 22305679

[23] Thyssen JP , Elias PM . Xerosis is latitude dependent and affects the propensity to develop atopic disease. J Allergy Clin Immunol. 2012;130(3):820‐821. doi:10.1016/j.jaci.2012.06.041 22841767

[24] Rodriguez A , Brickley E , Rodrigues L , Normansell RA , Barreto M , Cooper PJ . Urbanisation and asthma in low‐income and middle‐income countries: a systematic review of the urban–rural differences in asthma prevalence. Thorax. 2019;74(11):1020‐1030. doi:10.1136/thoraxjnl-2018-211793 31278168 PMC6860411

[25] Hanski I , von Hertzen L , Fyhrquist N , et al. Environmental biodiversity, human microbiota, and allergy are interrelated. Proc Natl Acad Sci USA. 2012;109(21):8334‐8339. doi:10.1073/pnas.1205624109 22566627 PMC3361383

[26] Yang Z , Zheng W , Yung E , Zhong N , Wong GWK , Li J . Frequency of food group consumption and risk of allergic disease and sensitization in schoolchildren in urban and rural China. Clin Exp Allergy. 2015;45(12):1823‐1832. doi:10.1111/cea.12532 25787117

[27] Ruokolainen L , Fyhrquist N , Haahtela T . The rich and the poor: environmental biodiversity protecting from allergy. Curr Opin Allergy Clin Immunol. 2016;16(5):421‐426. doi:10.1097/ACI.0000000000000304 27490122

[28] Luukkonen J , Moustgaard H , Martikainen P , Remes H . Does having siblings really protect against childhood atopic diseases? A total population and within‐family analysis. Eur J Epidemiol. 2024;39(3):289‐298. doi:10.1007/s10654-024-01104-w 38316709 PMC10995035

[29] Bager P , Wohlfart J , Westergaard T . Caesarean delivery and risk of atopy and allergic disesase: meta‐analyses. Clin Exp Allergy. 2008;38:634‐642. doi:10.1111/j.1365-2222.2008.02939.x 18266879

[30] Uekert S , Akan G , Evans M , et al. Sex‐related differences in immune development and the expression of atopy in early childhood. J Allergy Clin Immunol. 2006;118(6):1375‐1381. doi:10.1016/j.jaci.2006.09.008 17157669

[31] Social Insurance Institution . Lääkkeet ja lääkekorvaukset ‐ Erityiskorvaus ‐ 203 Krooninen keuhkoastma ja sitä läheisesti muistuttavat krooniset obstruktiiviset keuhkosairaudet . 203 Krooninen keuhkoastma ja sitä läheisesti muistuttavat krooniset obstruktiiviset keuhkosairaudet 2022 https://www.kela.fi/laake203

[32] Akdis CA . Does the epithelial barrier hypothesis explain the increase in allergy, autoimmunity and other chronic conditions? Nat Rev Immunol. 2021;21(11):739‐751. doi:10.1038/s41577-021-00538-7 33846604

[33] Akdis CA . The epithelial barrier hypothesis proposes a comprehensive understanding of the origins of allergic and other chronic noncommunicable diseases. J Allergy Clin Immunol. 2022;149(1):41‐44. doi:10.1016/j.jaci.2021.11.010 34822880

[34] Mäkinen TM , Juvonen R , Jokelainen J , et al. Cold temperature and low humidity are associated with increased occurrence of respiratory tract infections. Respir Med. 2009;103(3):456‐462. doi:10.1016/j.rmed.2008.09.011 18977127

[35] Murk W , Risnes KR , Bracken MB . Prenatal or early‐life exposure to antibiotics and risk of childhood asthma: a systematic review. Pediatrics. 2011;127(6):1125‐1138. doi:10.1542/peds.2010-2092 21606151

[36] Cichowicz R , Wielgosiński G , Fetter W . Dispersion of atmospheric air pollution in summer and winter season. Environ Monit Assess. 2017;189(12):605. doi:10.1007/s10661-017-6319-2 29103077 PMC5671516

[37] Huoneilman kosteus (Indoor humidity) . 2024. Accessed November 6, 2024. https://www.hengitysliitto.fi/kodin‐sisailma‐ja‐kunnossapito/sisailman‐laatu/sisailman‐olosuhteet/huoneilman‐kosteus/

[38] Schramm PJ , Brown CL , Saha S , et al. A systematic review of the effects of temperature and precipitation on pollen concentrations and season timing, and implications for human health. Int J Biometeorol. 2021;65(10):1615‐1628. doi:10.1007/s00484-021-02128-7 33877430 PMC9016682

[39] Lukkarinen M , Kirjavainen PV , Backman K , et al. Early‐life environment and the risk of eczema at 2 years—meta‐analyses of six Finnish birth cohorts. Pediatr Allergy Immunol. 2023;34(4):e13945. doi:10.1111/pai.13945 37102387

[40] Erbas B , Lowe AJ , Lodge CJ , et al. Persistent pollen exposure during infancy is associated with increased risk of subsequent childhood asthma and hayfever. Clin Exp Allergy. 2013;43(3):337‐343. doi:10.1111/cea.12071 23414542

[41] Ylianttila L , Visuri R , Hietanen M , Pastila R . Altistuminen UV‐säteilylle (Exposure to UV‐irradiation). Ultravioletti‐ ja lasersäteily (Ultraviolet and laser irradiation). Säteilyturvakeskus; 2009:203‐245.

[42] Frankel M , Bekö G , Timm M , Gustavsen S , Hansen EW , Madsen AM . Seasonal variations of indoor microbial exposures and their relation to temperature, relative humidity, and air exchange rate. Appl Environ Microbiol. 2012;78(23):8289‐8297. doi:10.1128/AEM.02069-12 23001651 PMC3497365

[43] Hui N , Parajuli A , Puhakka R , et al. Temporal variation in indoor transfer of dirt‐associated environmental bacteria in agricultural and urban areas. Environ Int. 2019;132:105069. doi:10.1016/j.envint.2019.105069 31400602

[44] Schoos AMM , Kragh M , Ahrens P , et al. Season of birth impacts the neonatal nasopharyngeal microbiota. Children. 2020;7(5):45. doi:10.3390/children7050045 32403236 PMC7278723

[45] Social Insurance Institution . Kela's Medicinal Products Database. 2022 https://asiointi.kela.fi/laakekys_app/LaakekysApplication?kieli=en

[46] Rubak S , Høst A , Christensen LB , Langfrits MS , Thomsen RW . Validity of asthma diagnoses and patterns of anti‐asthmatic drug use in a cohort of 2053 Danish children. Health Sci Rep. 2018;1:e77. doi:10.1002/hsr2.77 30623100 PMC6266370

