# Supplementary material for: Month of birth and outdoor temperature after birth predict childhood atopic diseases in Finland
Source: Pediatr Allergy Immunol. 2025 Jun 2;36(6):e70118. doi: 10.1111/pai.70118 (PMC12128010; doi:10.1111/pai.70118)
Supplement: Supplementary file 1 — Data S1. [file PAI-36-e70118-s001.docx]

**Supplementary Table S1** Medications for different atopic disease symptoms.

| Term in this study | Atopic disease symptoms | Medication | ATC codes |
| --- | --- | --- | --- |
| Antihistamines | Allergic reaction;  allergic rhinitis | Antihistamines for systemic use | R06 |
| Eczema medication | Atopic eczema;  allergic dermatitis | Corticosteroids for topical use | D07 |
| Asthma medication | Asthma | Inhaled corticosteroids and combination inhalers | R03BA01-08; R03BB04; R03BC01; R03BC03; R3DA05; R03DC01; R03DC03; R03AK03-13; R03AL02 |
| Epinephrine | Severe allergic reaction; anaphylaxis | Epinephrine injectors | C01CA24 |

**Supplementary Table S2** Prevalence of purchases of medication used for atopic diseases for children aged 0–15 years (%) by month of birth for the sibling population born during 1995–2004.

| Month of birth | N | Antihistamines |  | Eczema medication |  | Asthma medication |  | Epinephrine |  | Any  medication |
| --- | --- | --- | --- | --- | --- | --- | --- | --- | --- | --- |
| Jan | 28 340 | 36.0 |  | 30.7 |  | 17.4 |  | 2.7 |  | 54.1 |
| Feb | 26 262 | 36.7 |  | 30.5 |  | 17.6 |  | 2.9 |  | 54.2 |
| Mar | 30 007 | 36.4 |  | 30.2 |  | 17.4 |  | 2.8 |  | 54.3 |
| Apr | 29 688 | 36.1 |  | 29.9 |  | 17.2 |  | 2.4 |  | 54.2 |
| May | 29 402 | 35.2 |  | 29.8 |  | 16.5 |  | 2.4 |  | 53.6 |
| Jun | 28 893 | 34.4 |  | 30.0 |  | 16.3 |  | 2.2 |  | 52.8 |
| Jul | 30 067 | 34.1 |  | 30.4 |  | 16.3 |  | 2.3 |  | 53.3 |
| Aug | 29 233 | 34.3 |  | 31.5 |  | 17.0 |  | 2.3 |  | 54.1 |
| Sep | 28 515 | 34.2 |  | 31.2 |  | 17.2 |  | 2.6 |  | 53.4 |
| Oct | 27 719 | 33.9 |  | 31.7 |  | 17.2 |  | 2.7 |  | 53.8 |
| Nov | 25 373 | 34.7 |  | 31.2 |  | 17.4 |  | 2.7 |  | 53.8 |
| Dec | 25 958 | 35.2 |  | 30.8 |  | 17.5 |  | 2.8 |  | 53.6 |
|  |  |  |  |  |  |  |  |  |  |  |
| Total | 339 457 | 35.1 |  | 30.6 |  | 17.1 |  | 2.6 |  | 53.8 |
|  |  |  |  |  |  |  |  |  |  |  |
| max | min |  |  |  |  |  |  |  |  |  |

**Supplementary Table S3** Means and proportions (%) of observed confounders by month of birth for the full population born during 1995-2004.

| Month of birth | N | Girls, % | Mean birth  weight, grams | Mean gestational  age, days | Caesarean section,  % | Mean number of siblings | At least one parent born abroad, % | Rural residence,  % | Parental tertiary education, % | Parental above median income, % | Parental antihistamines, % | Parental eczema med., % | Parental asthma med., % | Parental Epinephrine, % |
| --- | --- | --- | --- | --- | --- | --- | --- | --- | --- | --- | --- | --- | --- | --- |
| Jan | 46 171 | 49.0 | 3 535 | 278 | 16.8 | 2.5 | 7.7 | 31.9 | 53.0 | 40.5 | 63.8 | 67.6 | 44.7 | 4.8 |
| Feb | 43 081 | 48.6 | 3 532 | 278 | 16.4 | 2.5 | 7.7 | 31.6 | 52.7 | 40.8 | 63.6 | 67.4 | 44.4 | 4.9 |
| Mar | 48 548 | 49.3 | 3 539 | 278 | 16.2 | 2.5 | 7.6 | 31.8 | 53.4 | 40.7 | 64.0 | 67.2 | 44.9 | 4.8 |
| Apr | 47 774 | 49.1 | 3 539 | 278 | 16.4 | 2.5 | 7.7 | 31.1 | 54.2 | 40.3 | 64.2 | 67.3 | 45.0 | 4.7 |
| May | 47 769 | 49.0 | 3 529 | 278 | 16.7 | 2.5 | 7.7 | 31.0 | 52.9 | 40.1 | 64.1 | 67.4 | 44.9 | 4.6 |
| Jun | 47 123 | 49.0 | 3 520 | 278 | 16.6 | 2.5 | 8.0 | 30.8 | 53.1 | 40.8 | 64.1 | 67.5 | 44.9 | 4.5 |
| Jul | 49 332 | 48.3 | 3 530 | 278 | 16.6 | 2.5 | 7.7 | 30.3 | 52.1 | 41.0 | 64.1 | 67.4 | 44.8 | 4.8 |
| Aug | 48 060 | 48.9 | 3 534 | 278 | 16.7 | 2.5 | 7.9 | 30.1 | 52.9 | 41.7 | 64.0 | 67.1 | 45.1 | 4.9 |
| Sep | 46 835 | 48.7 | 3 536 | 278 | 16.3 | 2.5 | 8.0 | 30.1 | 52.9 | 42.0 | 64.3 | 67.5 | 45.3 | 4.8 |
| Oct | 45 113 | 48.9 | 3 535 | 278 | 17.0 | 2.5 | 7.9 | 30.1 | 52.9 | 42.0 | 64.0 | 67.7 | 44.8 | 4.7 |
| Nov | 41 822 | 49.0 | 3 527 | 278 | 17.1 | 2.5 | 8.2 | 30.0 | 52.1 | 42.1 | 64.2 | 67.5 | 45.3 | 4.9 |
| Dec | 42 694 | 49.0 | 3 517 | 277 | 16.7 | 2.5 | 8.1 | 30.3 | 52.1 | 41.5 | 64.1 | 67.4 | 44.8 | 4.8 |
| Total | 554 322 | 48.9 | 3 531 | 278 | 16.6 | 2.5 | 7.8 | 30.8 | 52.9 | 41.1 | 64.1 | 67.4 | 44.9 | 4.8 |
|  |  |  |  |  |  |  |  |  |  |  |  |  |  |  |
| max | min |  |  |  |  |  |  |  |  |  |  |  |  |  |
|  |  |  |  |  |  |  |  |  |  |  |  |  |  |  |
| Number of siblings measured at age 15 years. Rural living is determined with urban-rural classification being rural at child's year of birth. Parental tertiary education and household income being above median measured | | | | | | | | | | | | | | |
| at child's year of birth. Parental medication purchases measured during the follow-up period of 15 years. | | | | | | | | | | | | | |  |

**Supplementary Table S4** Means and proportions (%) of observed confounders by month of birth for the sibling population born during 1995-2004.

| Month of birth | N | Girls, % | Mean birth  weight, grams | Mean gestational  age, days | Caesarean section,  % | Mean number of siblings | At least one parent born abroad, % | Rural residence,  % | Parental tertiary education, % | Parental above median income, % | Parental antihistamines, % | Parental eczema med., % | Parental asthma med., % | Parental Epinephrine, % |
| --- | --- | --- | --- | --- | --- | --- | --- | --- | --- | --- | --- | --- | --- | --- |
| Jan | 28 340 | 49.1 | 3 538 | 277 | 15.5 | 2.8 | 7.3 | 33.4 | 56.2 | 40.2 | 63.6 | 67.1 | 44.1 | 5.0 |
| Feb | 26 262 | 48.9 | 3 537 | 278 | 14.8 | 2.8 | 7.2 | 33.1 | 55.4 | 40.3 | 63.0 | 67.3 | 44.0 | 4.9 |
| Mar | 30 007 | 49.2 | 3 546 | 278 | 14.8 | 2.8 | 7.2 | 33.5 | 56.3 | 40.1 | 63.3 | 66.9 | 44.4 | 4.7 |
| Apr | 29 688 | 49.1 | 3 543 | 278 | 15.2 | 2.8 | 7.3 | 32.7 | 57.0 | 39.6 | 63.8 | 67.0 | 44.6 | 4.7 |
| May | 29 402 | 49.1 | 3 533 | 278 | 15.6 | 2.8 | 7.2 | 32.4 | 55.8 | 39.5 | 63.7 | 67.3 | 44.5 | 4.6 |
| Jun | 28 893 | 49.1 | 3 530 | 278 | 15.2 | 2.8 | 7.5 | 32.7 | 55.9 | 39.8 | 63.6 | 67.1 | 44.2 | 4.5 |
| Jul | 30 067 | 48.4 | 3 542 | 278 | 15.1 | 2.8 | 7.4 | 31.8 | 55.5 | 40.3 | 63.8 | 67.2 | 44.2 | 5.0 |
| Aug | 29 233 | 48.7 | 3 549 | 278 | 15.2 | 2.8 | 7.4 | 31.7 | 55.6 | 40.8 | 63.5 | 67.0 | 44.5 | 4.7 |
| Sep | 28 515 | 48.6 | 3 548 | 278 | 14.9 | 2.8 | 7.4 | 31.8 | 55.8 | 40.9 | 63.7 | 67.3 | 44.9 | 4.9 |
| Oct | 27 719 | 48.6 | 3 546 | 278 | 15.3 | 2.8 | 7.3 | 31.9 | 55.6 | 40.6 | 63.6 | 67.5 | 44.0 | 4.7 |
| Nov | 25 373 | 48.6 | 3 541 | 277 | 15.5 | 2.8 | 7.7 | 31.6 | 54.7 | 40.5 | 63.6 | 67.5 | 44.6 | 4.8 |
| Dec | 25 958 | 49.2 | 3 530 | 277 | 15.4 | 2.8 | 7.3 | 32.3 | 54.8 | 39.9 | 63.5 | 66.8 | 44.3 | 4.8 |
| Total | 339 457 | 48.9 | 3 540 | 278 | 15.2 | 2.8 | 7.3 | 32.3 | 55.7 | 40.2 | 63.6 | 67.2 | 44.4 | 4.8 |
|  |  |  |  |  |  |  |  |  |  |  |  |  |  |  |
| max | min |  |  |  |  |  |  |  |  |  |  |  |  |  |
|  |  |  |  |  |  |  |  |  |  |  |  |  |  |  |
| Number of siblings measured at age 15 years. Rural living is determined with urban-rural classification being rural at child's year of birth. Parental tertiary education and household income being above median measured | | | | | | | | | | | | | | |
| at child's year of birth. Parental medication purchases measured during the follow-up period of 15 years. | | | | | | | | | | | | | |  |

**Supplementary Figure S1** Moderation by outdoor temperatures after birth. Predicted probability (%) of purchases of antihistamines at ages 0–15 years by month of birth and 3-month average temperatures after birth. Results from full population models with 95% confidence intervals (N=551 531).


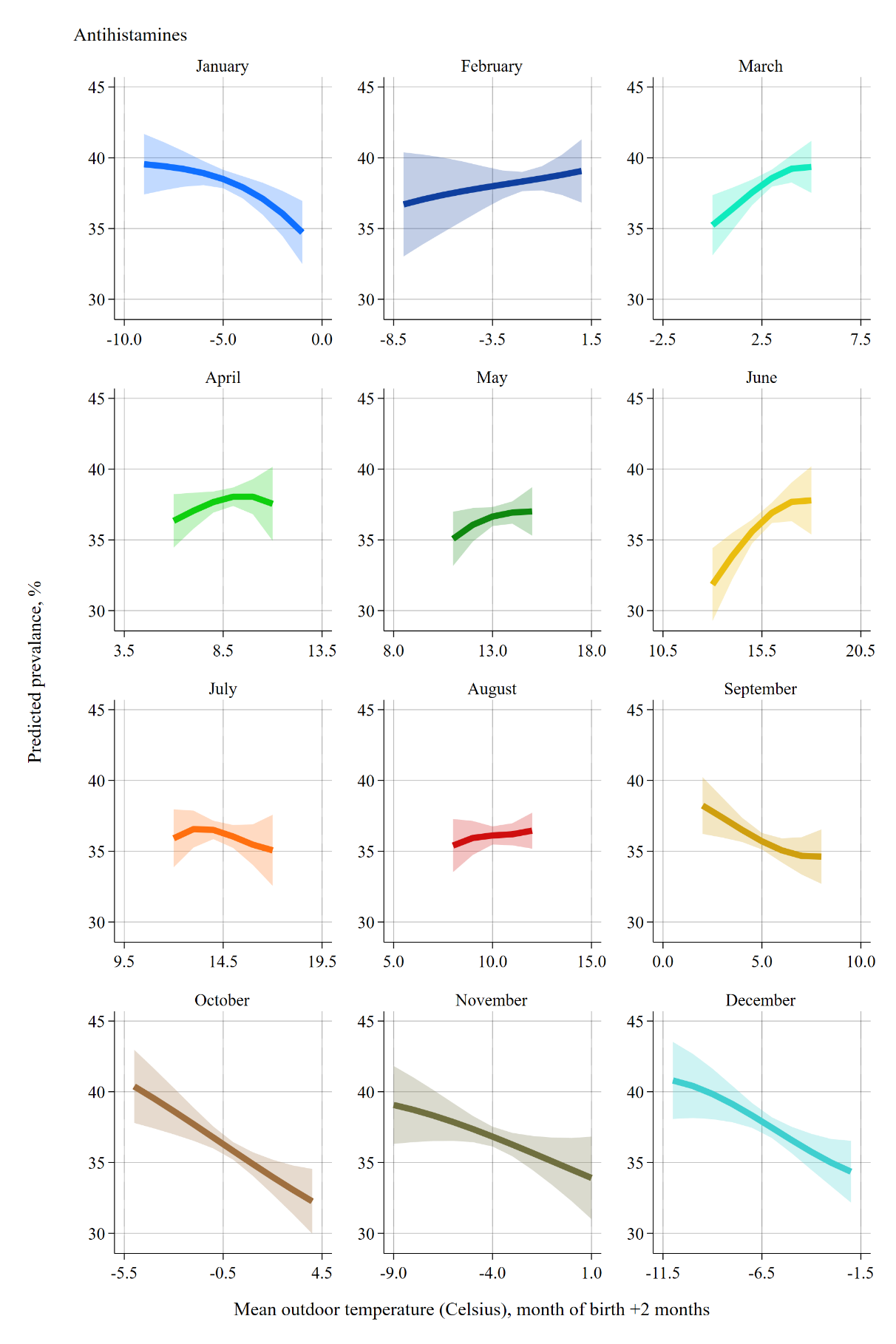


Each month of birth was analyzed separately with the 3-month averages of recorded outdoor temperatures in centigrade based on spatial and temporal variation during 1995–2004. Predictions for the values within the 90% interquantile range of temperatures for each month. Cubic polynomial of temperature was used. Note difference in x-axis values.

**Supplementary Figure S2** Moderation by outdoor temperatures after birth. Predicted probability (%) of purchases of eczema medication at ages 0–15 years by month of birth and 3-month average temperatures after birth. Results from full population models with 95% confidence intervals (N=551 531).


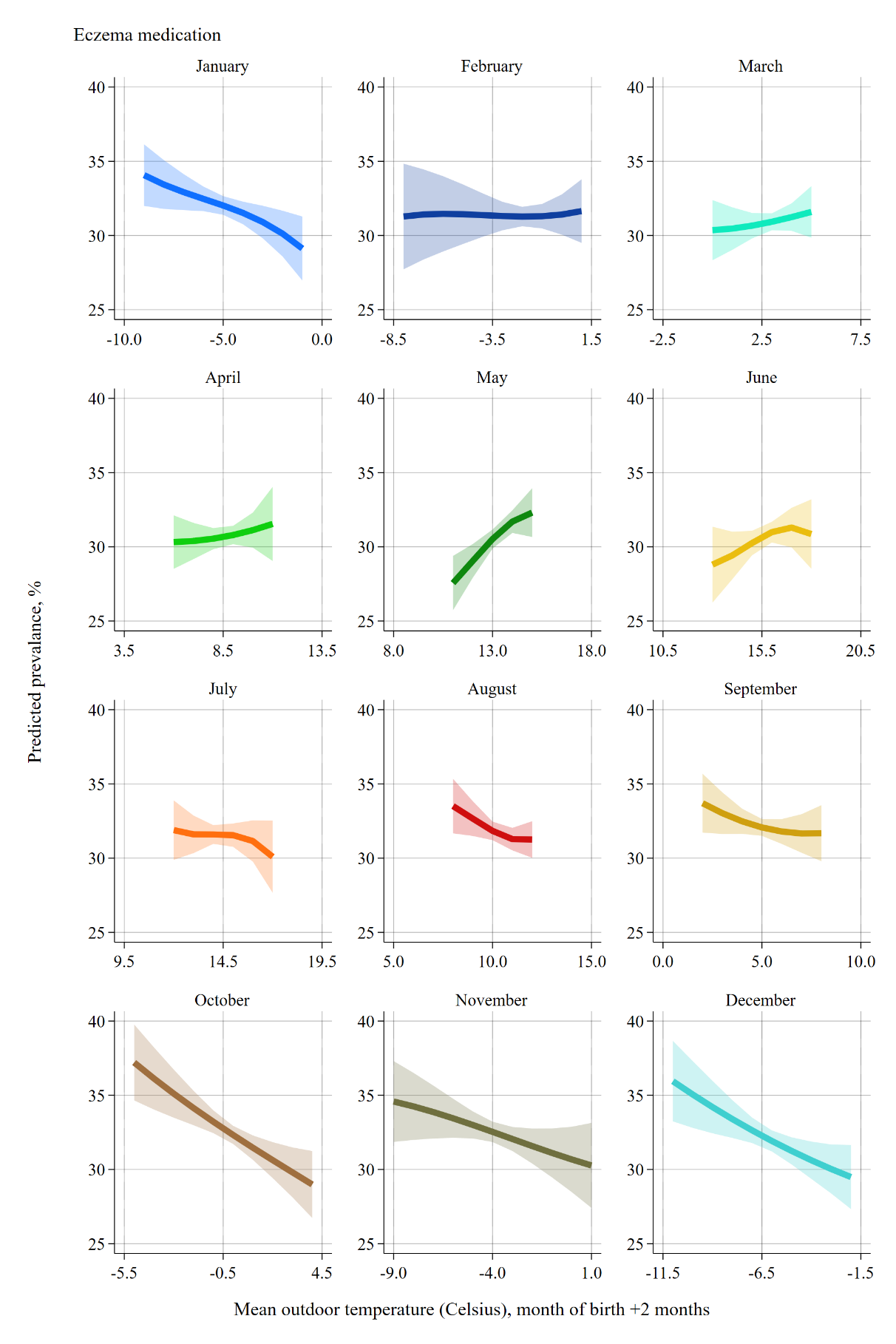


Each month of birth was analyzed separately with the 3-month averages of recorded outdoor temperatures in centigrade based on spatial and temporal variation during 1995–2004. Predictions for the values within the 90% interquantile range of temperatures for each month. Cubic polynomial of temperature was used. Note difference in x-axis values.

**Supplementary Figure S3** Moderation by outdoor temperatures after birth. Predicted probability (%) of purchases of asthma medication at ages 0–15 years by month of birth and 3-month average temperatures after birth. Results from full population models with 95% confidence intervals (N=551 531).


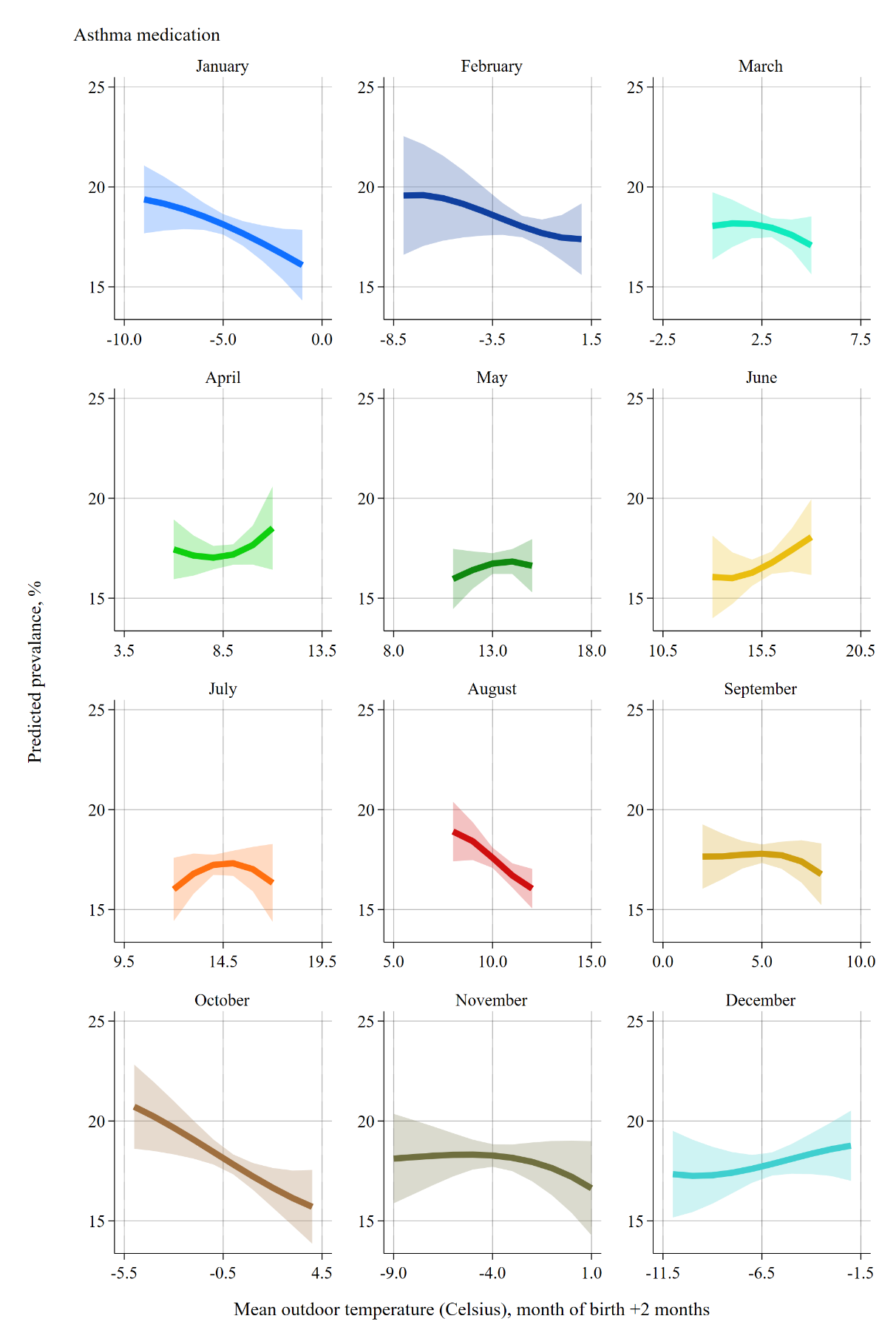


Each month of birth was analyzed separately with the 3-month averages of recorded outdoor temperatures in centigrade based on spatial and temporal variation during 1995–2004. Predictions for the values within the 90% interquantile range of temperatures for each month. Cubic polynomial of temperature was used. Note difference in x-axis values.

**Supplementary Figure S4** Moderation by outdoor temperatures after birth. Predicted probability (%) of purchases of epinephrine at ages 0–15 years by month of birth and 3-month average temperatures after birth. Results from full population models with 95% confidence intervals (N=551 531).


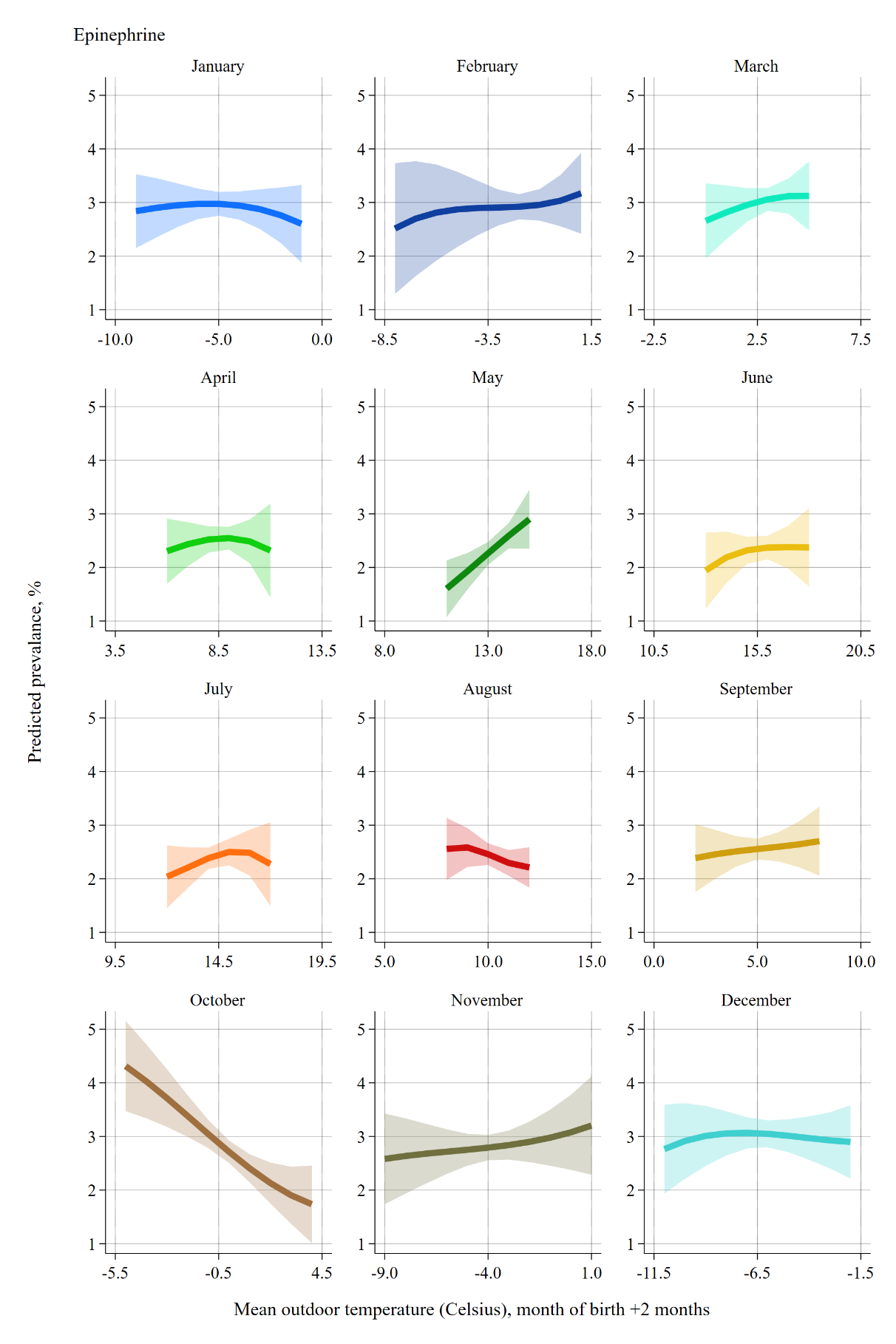


Each month of birth was analyzed separately with the 3-month averages of recorded outdoor temperatures in centigrade based on spatial and temporal variation during 1995–2004. Predictions for the values within the 90% interquantile range of temperatures for each month. Cubic polynomial of temperature was used. Note difference in x-axis values.

**Supplementary Figure S5** Predicted prevalence (%) for purchases of medication used for atopic diseases at ages 0–15 years by month of birth in reference to January. Moderation by sex. Results from fully adjusted full population models (N=554 322) with 95% confidence intervals.


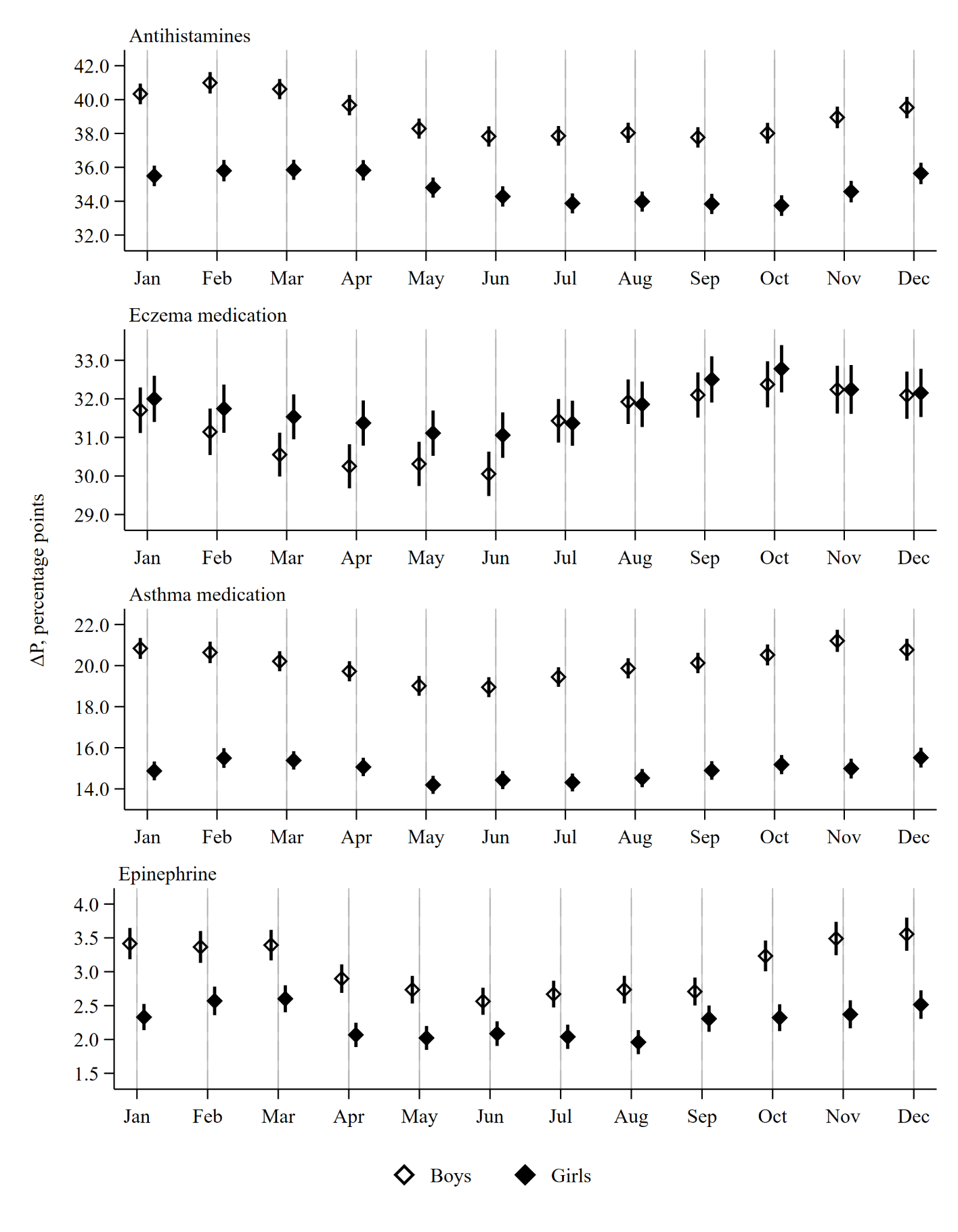


The model is adjusted for child’s sex, birth year, and immigrant background; region and urbanicity of residence; household income and parental education; sibship size and birth order; mode of delivery, gestational age (squared) and birth weight (squared); and parental medication purchases. The sibling fixed-effects model is adjusted in the same way as the fully adjusted model with the addition of sibship-specific dummy variables.

**Supplementary Figure S6** Estimated difference in probability (∆P, percentage points) of at least three purchase years for medication used for atopic diseases and special reimbursement entitlement for asthma medication at ages 0–15 years by month of birth in reference to January. Results from A) full population models (N=554 322) and B) sibling population models (N= 339 457) with 95% confidence intervals.


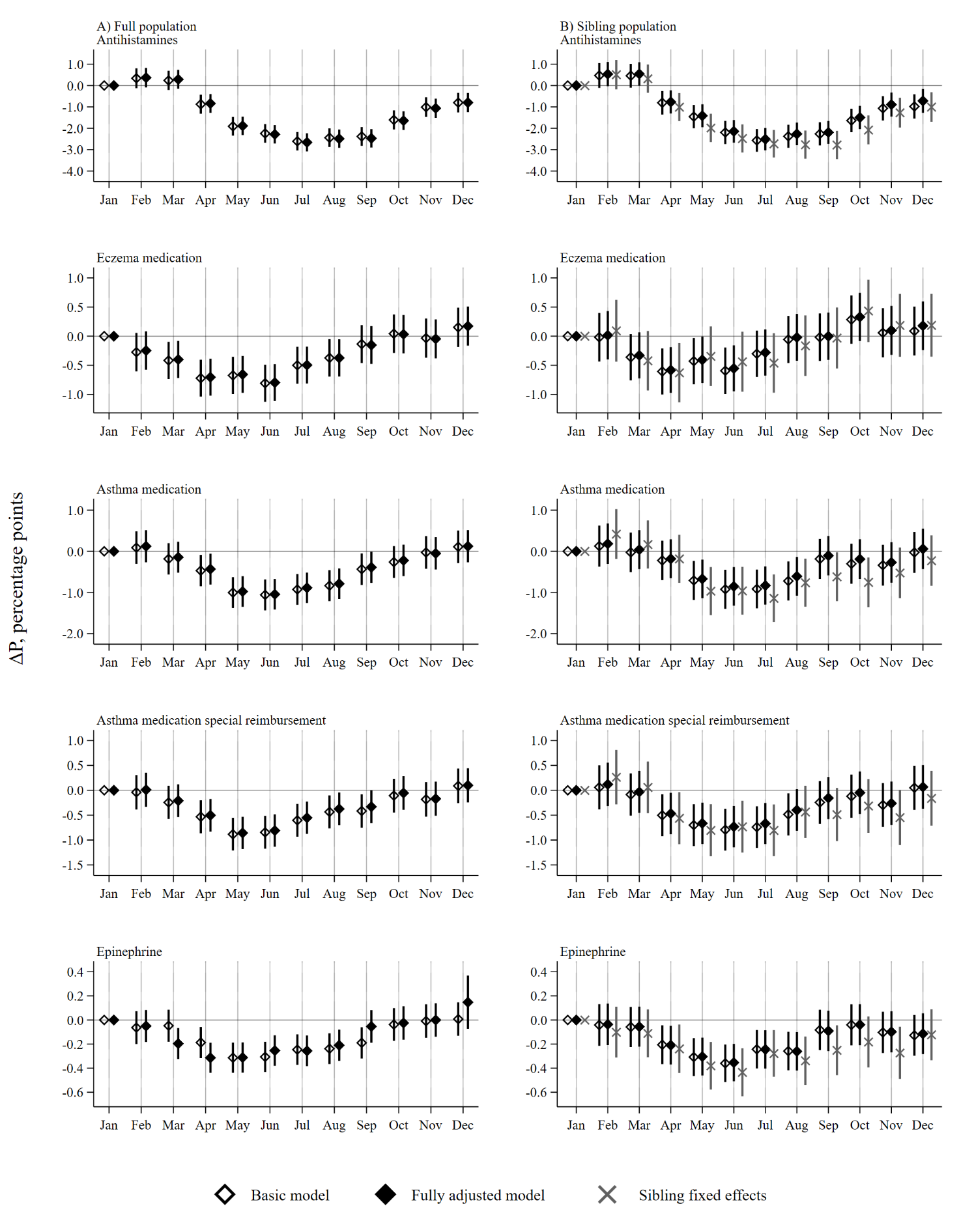


The basic model is adjusted for child’s sex, birth year, and immigrant background. The fully adjusted model is further adjusted for region and urbanicity of residence; household income and parental education; sibship size and birth order; mode of delivery, gestational age (squared) and birth weight (squared); and parental medication purchases. The sibling fixed-effects model is adjusted in the same way as the fully adjusted model with the addition of sibship-specific dummy variables.

**Supplementary Figure S7** Moderation by outdoor temperatures after birth. Predicted probability (%) of at least three purchase years for antihistamines by month of birth and 3-month average temperatures after birth. Results from full population models with 95% confidence intervals (N=551 531).


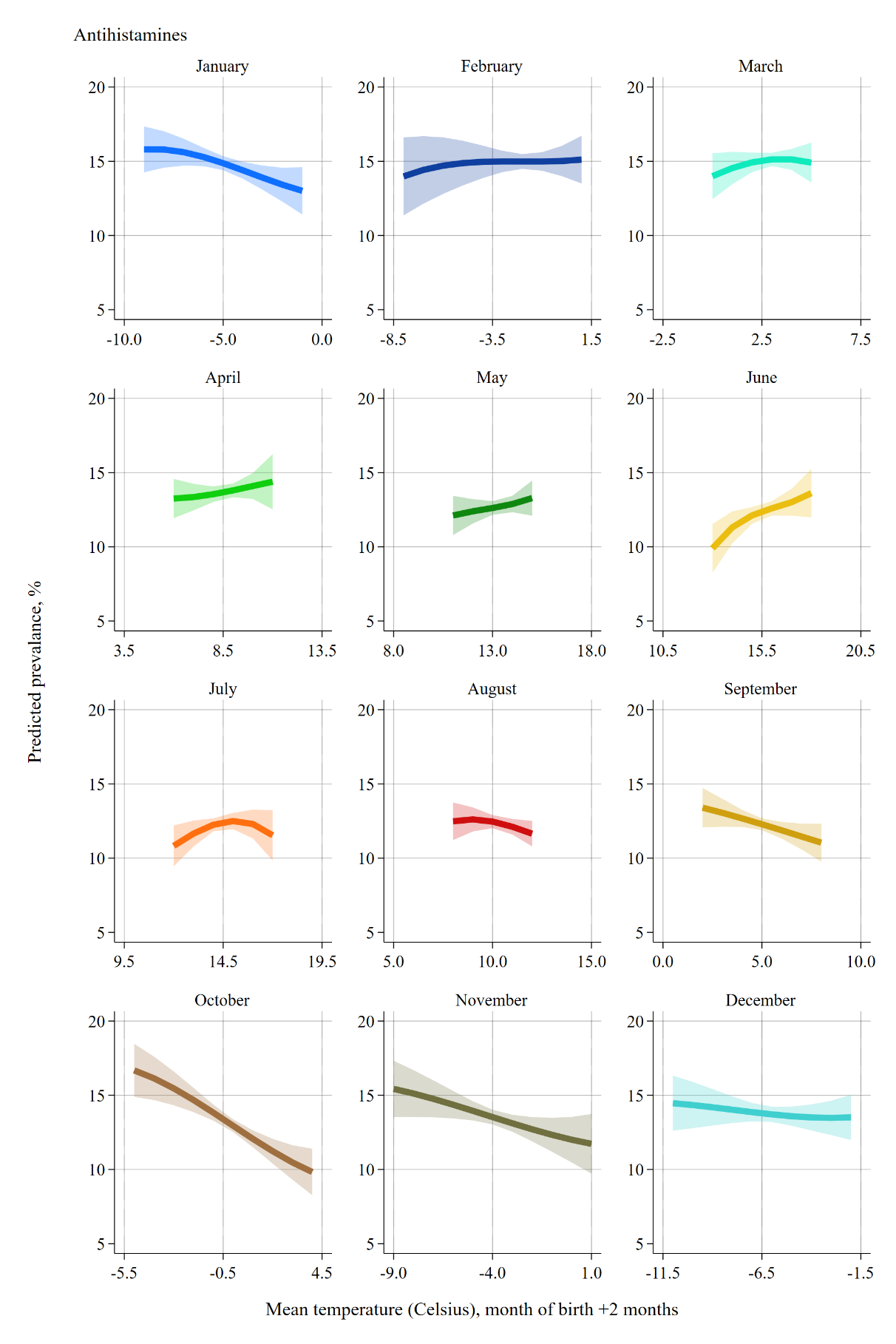


Each month of birth was analyzed separately with the 3-month averages of recorded outdoor temperatures in centigrade based on spatial and temporal variation during 1995–2004. Predictions for the values within the 90% interquantile range of temperatures for each month. Cubic polynomial of temperature was used. Note difference in x-axis values.

**Supplementary Figure S8** Moderation by outdoor temperatures after birth. Predicted probability (%) of at least three purchase years for eczema medication by month of birth and 3-month average temperatures after birth. Results from full population models with 95% confidence intervals (N=551 531).


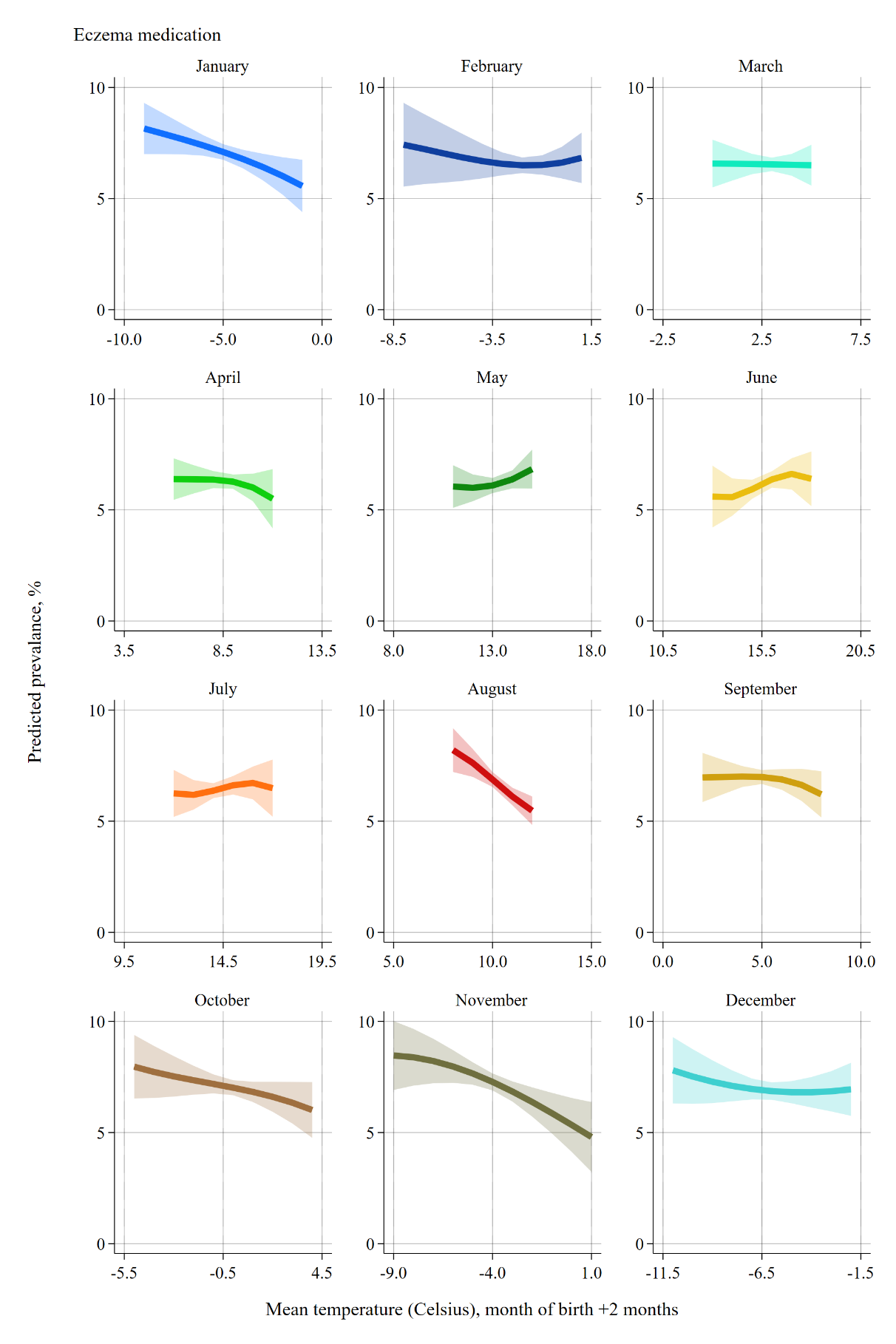


Each month of birth was analyzed separately with the 3-month averages of recorded outdoor temperatures in centigrade based on spatial and temporal variation during 1995–2004. Predictions for the values within the 90% interquantile range of temperatures for each month. Cubic polynomial of temperature was used. Note difference in x-axis values.

**Supplementary Figure S9** Moderation by outdoor temperatures after birth. Predicted probability (%) of at least three purchase years for asthma medication by month of birth and 3-month average temperatures after birth. Results from full population models with 95% confidence intervals (N=551 531).


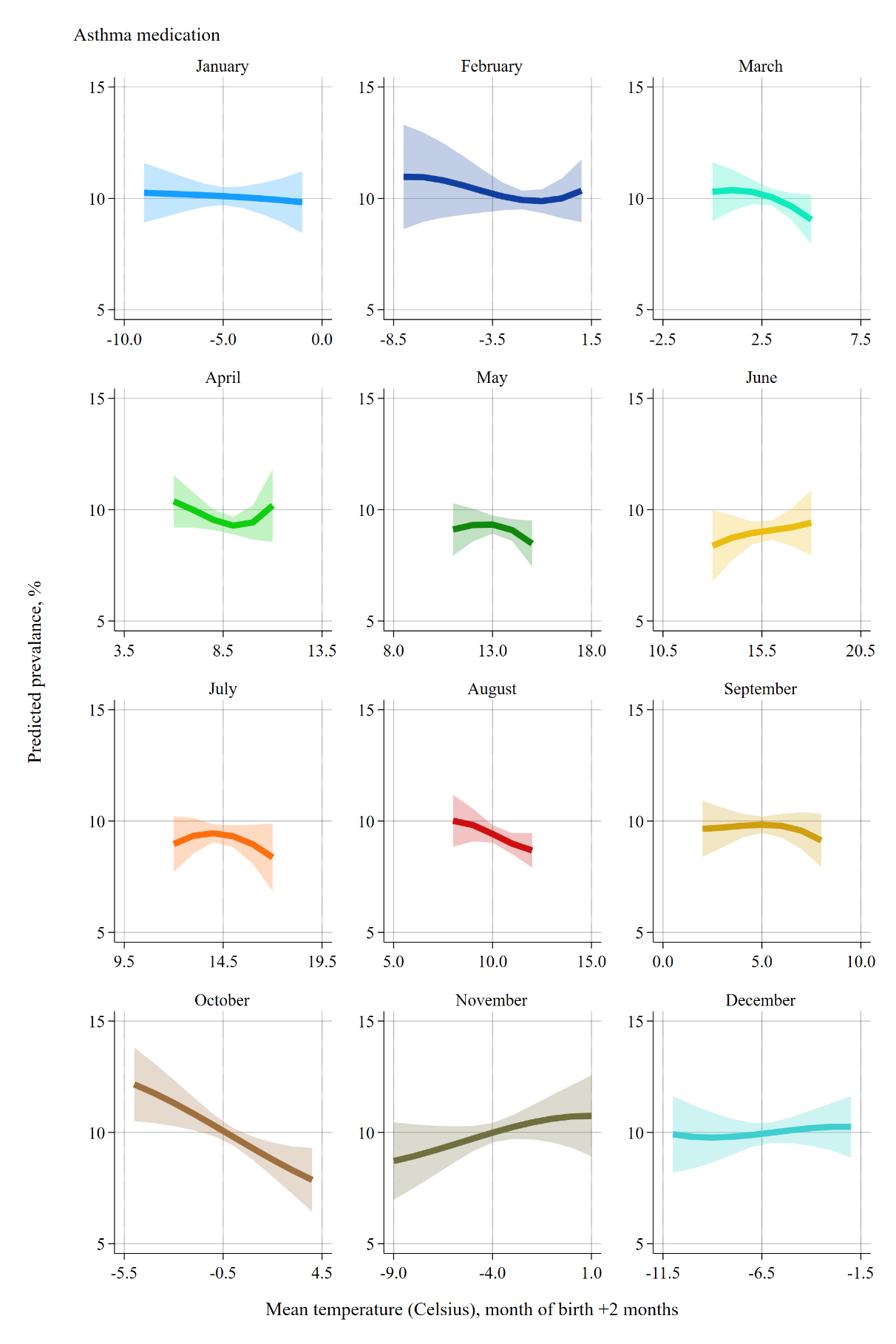


Each month of birth was analyzed separately with the 3-month averages of recorded outdoor temperatures in centigrade based on spatial and temporal variation during 1995–2004. Predictions for the values within the 90% interquantile range of temperatures for each month. Cubic polynomial of temperature was used. Note difference in x-axis values.

**Supplementary Figure S10** Moderation by outdoor temperatures after birth. Predicted probability (%) of special reimbursement entitlement for asthma medication by month of birth and 3-month average temperatures after birth. Results from full population models with 95% confidence intervals (N=546 207).


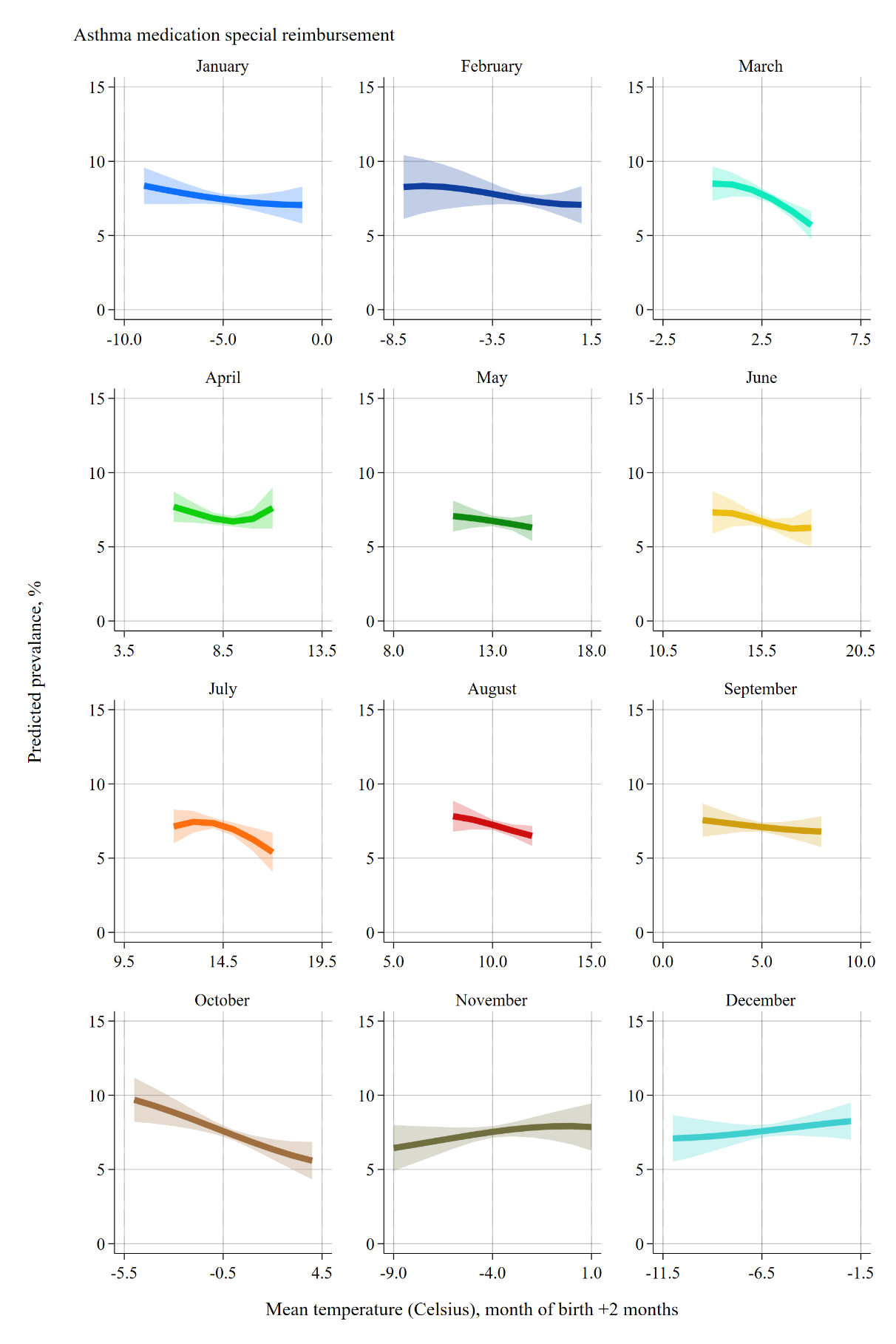


Each month of birth was analyzed separately with the 3-month averages of recorded outdoor temperatures in centigrade based on spatial and temporal variation during 1995–2004. Predictions for the values within the 90% interquantile range of temperatures for each month. Cubic polynomial of temperature was used. Note difference in x-axis values.

**Supplementary Figure S11** Moderation by outdoor temperatures after birth. Predicted probability (%) of at least three purchase years for epinephrine by month of birth and 3-month average temperatures after birth. Results from full population models with 95% confidence intervals (N=546 207).


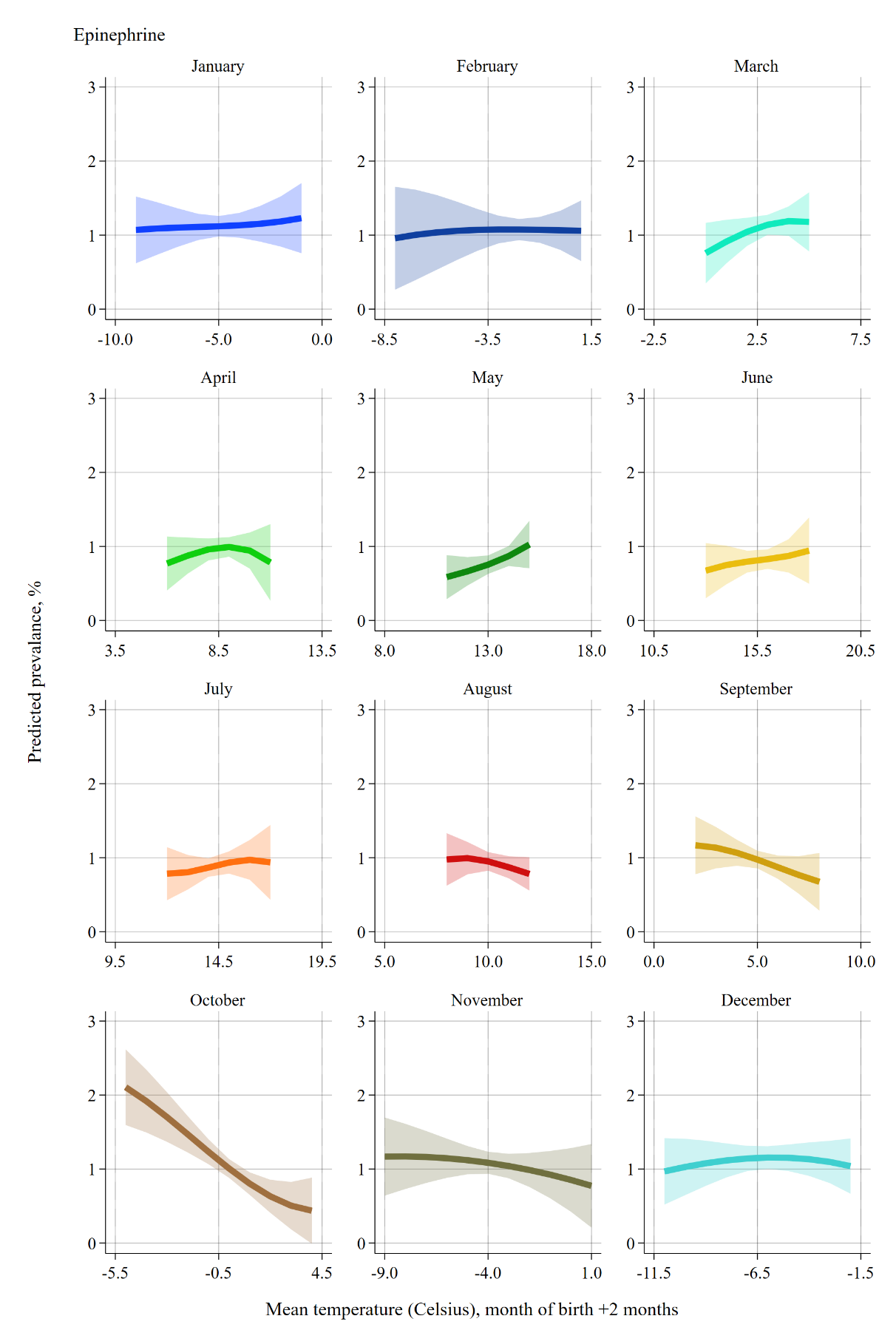


Each month of birth was analyzed separately with the 3-month averages of recorded outdoor temperatures in centigrade based on spatial and temporal variation during 1995–2004. Predictions for the values within the 90% interquantile range of temperatures for each month. Cubic polynomial of temperature was used. Note difference in x-axis values.

**Supplementary Figure S12** Moderation by outdoor temperatures after birth. Predicted probability (%) of any purchases of medication used for atopic diseases at ages 0–15 years by month of birth and average outdoor temperatures for 1- to 3-month periods after birth. Results from full population models with 95% confidence intervals (N=551 531). Confidence intervals not shown due to sake of clarity.


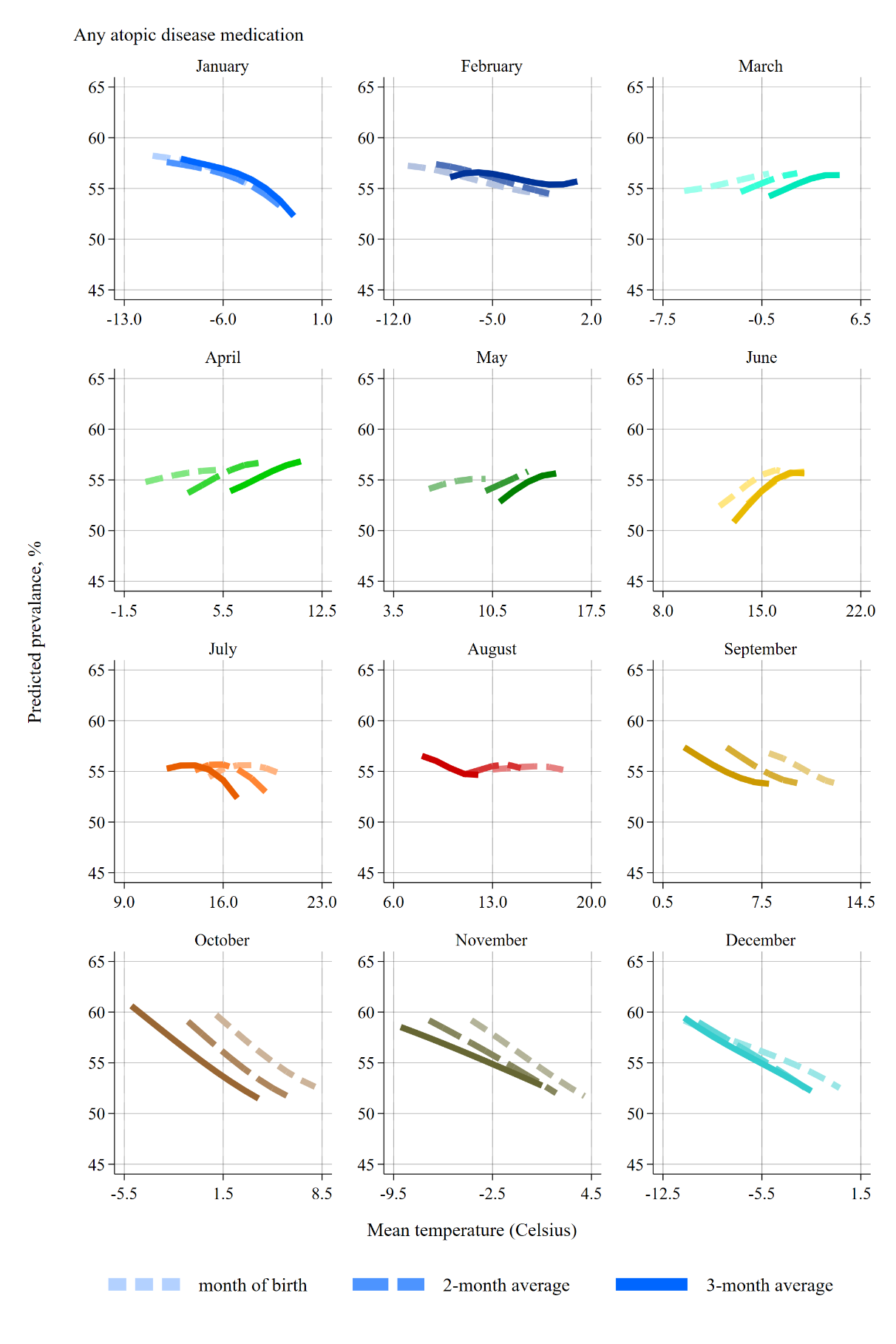


Each month of birth was analyzed separately with the averages of recorded outdoor temperatures in centigrade based on spatial and temporal variation during 1995–2004. Predictions for the values within the 90% interquantile range of temperatures for each month. Cubic polynomial of temperature was used. Note difference in x-axis values.
